# *De novo* transcriptome assembly of the lobster cockroach *Nauphoeta cinerea* (Blaberidae)

**DOI:** 10.1590/1678-4685-GMB-2017-0264

**Published:** 2018-07-16

**Authors:** Ana Lúcia Anversa Segatto, José Francisco Diesel, Elgion Lucio Silva Loreto, João Batista Teixeira da Rocha

**Affiliations:** 1 Departamento de Bioquímica e Biologia Molecular, CCNE, Universidade Federal de Santa Maria, Santa Maria, RS, Brazil

**Keywords:** Nauphoeta cinerea, transcriptome assembly, fat body, detoxification

## Abstract

The use of *Drosophila* as a scientific model is well established, but the use of cockroaches as experimental organisms has been increasing, mainly in toxicology research. *Nauphoeta cinerea* is one of the species that has been studied, and among its advantages is its easy laboratory maintenance. However, a limited amount of genetic data about *N. cinerea* is available, impeding gene identification and expression analyses, genetic manipulation, and a deeper understanding of its functional biology. Here we describe the *N. cinerea* fat body and head transcriptome, in order to provide a database of genetic sequences to better understand the metabolic role of these tissues, and describe detoxification and stress response genes. After removing low-quality sequences, we obtained 62,121 transcripts, of which more than 50% had a length of 604 pb. The assembled sequences were annotated according to their genes ontology (GO). We identified 367 genes related to stress and detoxification; among these, the more frequent were p450 genes. The results presented here are the first large-scale sequencing of *N. cinerea* and will facilitate the genetic understanding of the species' biochemistry processes in future works.

## Introduction

The understanding of biological phenomena is dependent on the organisms observed, and increasing the variety of experimental organisms can provide clearer views of the targeted processes (Golstein *et al.*, 2003). Among the reasons that have motivated the search for alternative experimental organisms is also the pressure to reduce the use of mammalian species in toxicological testing (Peterson *et al.*, 2008). As an alternative experimental organism, the use of the cockroach is increasing (Walz *et al.*, 2006; Blankenburg *et al.*, 2015). Cockroaches (order Dictyoptera) are primitive winged insects, with worldwide distribution and comprising approximately 4,000 species (Bell *et al.*, 2007). Thirty of these species are associated with human household, and less than 1% is thought to be associated with human health problems (Fakoorziba *et al.*, 2010).

*Periplaneta americana* (L.) is the most common domestic cockroach species, it shows great reproductive capability and has been used as an experimental organism in scientific studies, including neurophysiology research (Wicher *et al.*, 2006; Nishino *et al.*, 2010). At least three transcriptomes of different *P. americana* tissues were published (Chen *et al.*, 2015; Zhang *et al.*, 2016; Kim *et al.*, 2016). Another cockroach species frequently used as scientific experimental organism is the German cockroach *Blattella germanica* (L.) (Cruz *et al.*, 2006). This species is highly dependent on humans for survival, and its domestic presence is associated with the occurrence of allergic respiratory diseases (Brenner, 1995). One transcriptome of this species has been published (Zhou *et al.*, 2014).

The lobster cockroach *Nauphoeta cinerae* (Olivier) has been used as an experimental model for toxicology (Rodrigues *et al.*, 2013; Adedara *et al.*, 2015, 2016) and shown to be a valid alternative for basic toxicological studies. Compared with the other species of cockroaches used in scientific studies, *N. cinerea* is easy to handle and does not fly, facilitating its maintenance in the laboratory. However, until now, there are few genetic sequences available for this species, and studies using molecular biology techniques like quantitative real-time PCR have been laborious to implement.

Cockroaches are found in diverse and inhospitable habitats that can have different amounts of toxic substances, such as environmental pollutants, microbial toxins, insecticides, and other xenobiotics. Thus, they may be a good experimental model to study detoxification abilities and stress response (Bell *et al.*, 2007; Zhang *et al.*, 2016). The usual response to stress conditions is the overproduction of reactive oxygen species (ROS), resulting in redox homeostasis alterations as well as oxidative stress. Overproduction of ROS have been associated to the toxicity of a wide range of xenobiotics, such as benzo[a]pyrene (Winn and Wells 1997), methamphetamine (McCallum *et al.*, 2011; Wong *et al.*, 2008), ethanol (Dong*et al.*, 2008, 2010), sodium fluoride (Umarani *et al.*, 2015; Samanta *et al.*, 2016; Song *et al.*, 2017), and methylmercury (Usuki and Fujimura, 2016). However, ROS are also produced by normal cellular metabolism, and one of its beneficial effects is on the organism's defense system (Valko *et al.*, 2007).

The main components of the antioxidant system are conserved along the evolutionary process, but there are different adaptations in different groups. In insects, the major change in comparison to other phylogenetic groups is the absence of selenium-dependent glutathione peroxidase (SeGPx). It has been proposed that in insect GPxs evolution, selenium was replaced by cysteine more than once (Bae *et al.*, 2009; Flohe *et al.*, 2011). Due the variations among groups, the detoxification genes being expressed should be known before starting studies of exposure to toxic compounds.

Antioxidant enzymes can be divided as acting in phase I (primary) and phase II (secondary) reactions. Phase I reactions consist of oxidation, hydrolysis and reduction, and the enzymes involved are aldehyde dehydrogenases, alcohol dehydrogenases, catalases, cytochrome P450s, dehalogenases, hydroxylases, oxidoreductases, peroxidases, superoxide dismutases (SODs), thioredoxins, and glutaredoxins. Secondary antioxidant enzymes that act indirectly on ROS include acetyltransferases, acyltransferase, and glutathione S-transferases (GSTs).

The fat body, together with midgut, is the primary detoxification organ in insects, but its gene expression profile is poorly addressed. In addition, the fat body plays an essential role in most intermediary metabolism reactions, as well as energy storage and utilization (Arresse and Soulages, 2010), besides being an endocrine organ producing several antimicrobial peptides (Wen *et al.*, 2011).

Genetic and transcriptome information of *N. cinerae* cockroaches is scarce. Transcriptome sequencing is a cost-effective tool for non-model organisms and the high-throughput sequencing technologies are an efficient method for genomic characterization of a species and gene discovery (Wang *et al.*, 2009). Here, we describe the transcriptome of *N. cinerae* fat body and head, with a focus on the detoxification and stress response genes. We also analyzed the differential gene expression of tissues to investigate the metabolic activity of the fat body and its participation in detoxification routes.

## Materials and Methods

### Cockroach rearing, RNA isolation and Illumina sequencing

The *N. cinerea* specimens used in this work were obtained from the Laboratório de Bioquímica Toxicologica, Universidade Federal de Santa Maria, Brazil. The insects were maintained in dark plastic boxes under standard conditions (Adedara *et al.*, 2015). RNA was extracted from two dissected body parts: the head (all its tissues, including the central nervous system), and the fat body (containing epidermal, neuronal, muscle cells, and other integument constituents). Two and four individuals were used for head and fat body RNA extraction, respectively. Total RNA was prepared from the two tissues using Trizol Reagent (Life Technologies) according to the manufacturer's instructions. A second extraction procedure was done from the product of the first for RNA cleanup. Library preparation followed the recommendation of the TruSeq^®^ Stranded mRNA Library Prep Kit (Illumina). Briefly, total RNA was purified and fragmented. The cleaved RNA fragments were then employed for first-strand cDNA synthesis, followed by second-strand cDNA synthesis. Fragments underwent a 3' adenylation process and were ligated with adapters before the PCR reaction was carried out. After library validation, the products were normalized and pooled. The MiSeq Reagent Kit v3 (150 cycles) was employed for sequence in a Illumina MiSeq system, resulting in pair-end reads of 75 pb. We used 25% of the flow cell for each sample. Sequencing was carried out using an Illumina MiSeq Plataform by Unidade de Genôminca Computacional Darcy Fontoura de Almeida/LNCC/Brazil.

### Read processing, *de novo* transcriptome assembly, and annotation

Read quality was checked and visualized using FastQC v 0.11.4 (Andrews, 2010), and low quality reads and adapters were eliminated using Trim Galore v 0.4.1 (Wu *et al.*, 2011). Reads with a quality threshold of less than 25 on the Phred scale, empty reads, and short sequences with a length of less than 25 nt were removed. The *de novo* assembly was performed with Trinity v 2.2.0 software using default settings (Grabherr *et al.*, 2011). The Deconseq Standalone v 0.4.3 program (Schmieder and Edwards, 2011) was used to remove contamination from virus, bacteria, and human sequences, and contigs with such hits were removed from the assembly before further analysis. We performed three assemblies: one from head reads, one from fat body reads, and one combining all the reads. The latter was used as a reference in the transcript quantification and differential expression analysis. Functional annotation was performed using Trinotate v 3.0.1 (Haas *et al.*, 2013) with a cutoff E-value of 10^-5^. CateGOrizer v 3.21 (Hu *et al.*, 2008) was used to map GO terms to GO Slim file by single count in order to get a broad overview of the functional classification of the transcripts. Each GO term was manually assigned to a consensus functional class. The assembly quality was assessed by examining the percentage of input RNA-Seq reads that are represented in the transcriptome assembly. The representation of full-length reconstructed protein-coding genes was evaluated by searching the assembled transcripts against SwissProt (E-value 10^-20^) and calculating the values of Nx statistics. Also, in order to verify the quality of our transcriptome and the similarity among transcripts of different species of cockroaches, we used our reference transcriptome as query and performed a blastn search using *P. americana* (Chen *et al.*, 2015) or *B. germanica* (Zhou *et al.*, 2014) transcriptomes as target database. To this end, we downloaded the raw reads of *P. americana* and *B. germanica* and used Trinity v 2.2.0 (Grabherr *et al.*, 2011) for the assembly, just as done for our data. The online tool BioVenn (Hulsen *et al.*, 2008) was used to draw a Venn diagram showing the distribution of transcripts detected in the head and fat body by a blastx, with a cutoff E-value of 10^-5^, using the UniProt database as subject (The UniProt Consortium, 2017).

### Transcript quantification and differential expression analysis

The assembly with reads from the head and fat body was used as a reference to estimate transcript abundance and build a counts matrix for each of the transcripts in each tissue using RSEM v 1.2.27 software (Li and Dewey, 2011). The expression abundance of isoforms was normalized using the TPM (transcripts per million) method (Wagner *et al.*, 2012). Differential expression analyses were carried out using the dgeR Bioconductor package (Robinson *et al.*, 2010) using a false discovery rate (FDR) cutoff of 0.001. Isoforms up-regulated in each sample were isolated and analyzed using Perl scripts and annotation was carried out using Blast2GO (Conesa *et al.*, 2005) using a cutoff E-value of 10^-5^. CateGOrizer v 3.21 (Hu *et al.*, 2008) was used to map GO terms to GO Slim file by single count.

### Identification and analysis of genes of interest

To directly identify detoxification and stress response genes in our transcriptome, we translated our transcripts in the six frames using the EMBOSS Transeq command line (Rice *et al.*, 2000; Goujon *et al.*, 2010), and used this as a database in blast+ using blastx (Camacho *et al.*, 2008). We searched for genes related to oxidation and reduction, conjugation, hydrolysis, and other functions possibly related to stress (Xu *et al.*, 2013) in Flybase to then be used as a query.

## Results

### *De novo* assembly of an *N. cinerea* transcriptome

A total of 24,980,364 Illumina MiSeq reads from fat body and head tissue were generated. The SRA submission reads can be found under the accession numbers SRR3581673 and SRR3581312. After trimming, 23,137,682 reads were assembled in a reference transcriptome, combining head and fat body reads, this resulting in 62,121 transcripts with an N50 of 604 (Table 1). The separated assemblies of the head and fat body transcriptomes (Table 1) were used in transcript quantification and the differential expression analysis. To assess the quality of our assembly we also mapped the reads back to the transcripts and found that among 70-80% of the fragments were mapped as proper pairs. In each assembly, approximately 2,000 transcripts are represented by nearly full-length proteins, having > 80% alignment coverage.

**Table 1:** Summary statistics of transcriptome assembly.

	Head	Fat body	Reference
Number of reads	12,935,304	12,045,060	24,980,364
Reads after trimming	6,098,525	5,470,316	23,137,682
Total Trinity genes	41,631	27,568	57,928
Total Trinity transcripts	43,991	29,163	62,121
Percent CG	38.78	37.94	38.38
Total assembled bases	21,525,406	13,939,303	31,375,798
Transcript contig N50	576	547	604

### Functional annotation of the reference transcriptome

**Figure 1:**
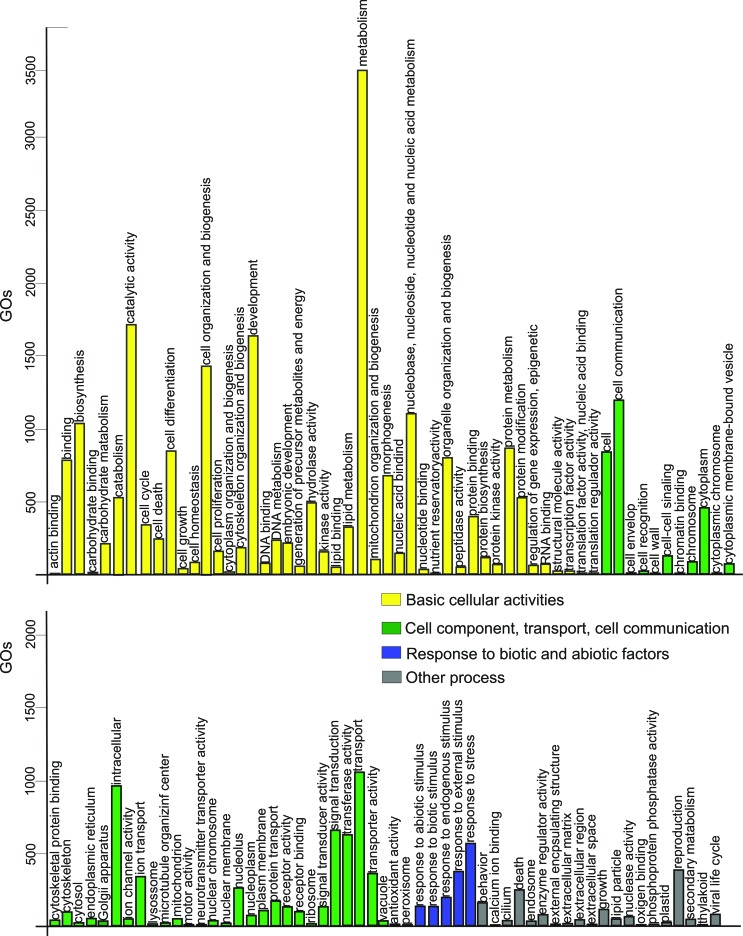
GO slim terms distribution associated with the *N. cinerea* transcriptomes.

The transcriptome was annotated using Trinotate v3.0.1 (Haas *et al.*, 2013). Blast homologies were captured by searching the UniProt/Swiss-Prot protein databases for further GO assignments. Mapping Entrez Gene IDs to GO annotations identified 44,963 terms categorized in 114 functional groups. The GO terms attributed to the greatest number of genes, in descending order, were metabolism (GO:0008152), catalytic activity (GO:0003824), and development (GO:0007275) (Figure 1). Twelve GOs were classified with antioxidant activity and 558 as response to stress activity. The assembly of *P. americana* resulted in 158,464 transcripts with a N50 of 536 and the assembly of *B. germanica* resulted in 71,903 transcripts with a N50 of 904*.* Our assembly strategy resulted in more transcripts and a higher N50 for *B. germanica* than previously described (Zhou *et al.*, 2014). For *P. americana*, our assembly strategy resulted in more transcripts but with a smaller average size than that previously described (Chen *et al.*, 2015). A total of 533 *N. cinerea* transcripts showed a Blast similarity with *P. americana,* and 2,679 with *B. germanica* with an E-value of zero. The top 10 hits of blastn results, with lower E-value and higher bit score are listed in Supplementary Information Table S1. The percentage of GC was similar in all the cockroach transcriptomes assembled, being, near 37.94 in *N. cinerea* fat body and 40.08 in *P. americana* testis.

### Differential expression

**Figure 2:**
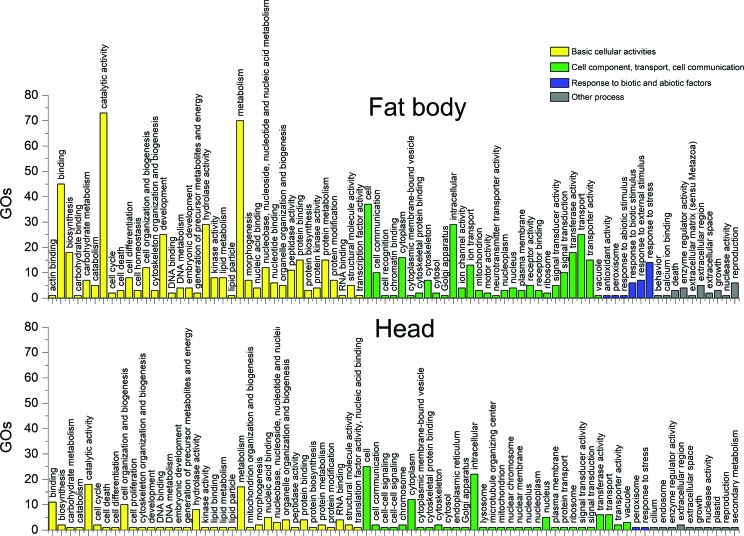
GO slim terms distribution of upregulated genes in the fat body and head of *N. cinerea.*

**Figure 3:**
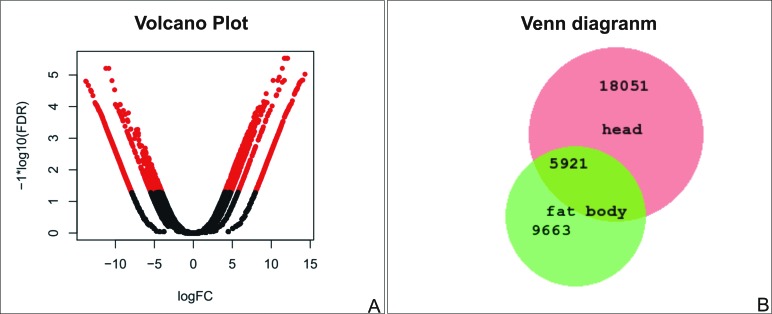
Comparison among head and fat body transcripts. (A) Volcano plot of differentially expressed transcripts; the X-axis displays the fold change expression differences (FC) and the Y-axis the statistical significance based on a false discovery rate (FDR) cut-off of 0.001. (B) Venn diagram of blastx results of transcripts detected in the head and fat body.

Using a dispersion of 0.1 in edgeR (Robinson *et al.*, 2010) we found 3,257 isoforms as differentially expressed in the head and fat body of the lobster cockroach. The up-regulated isoforms in the head were classified in 1,749 GO terms and could be categorized into 77 functional groups. In the fat body, a total of 864 GO terms were categorized into 81functional groups (Figure 2). Regarding species distribution, the top hit species was *Zootermopsis nevadensis* Hagenfor. *Blattella germanica,* and *P. americana* appeared among the six top hits in both tissues. Among the classification of up-regulated genes in the fat body, 30 GOs were associated with response to biotic and abiotic factors, while only 10 up-regulated genes were found in the head in this subcategory. Differentially expressed transcripts are shown in red in the volcano plot of Figure 3A. From the total of 43,991 transcripts assembled in the head and 29,163 assembled in the fat body, 37,743 and 24,174 had blastx results respectively, using the UniProt database as subject and an E-value of 10^-5^ as cut-off. After removing redundant hits, 5,921 Blast results were common between the assembled transcriptomes (Figure 3B).

### Detoxification and stress genes

We identified 367 transcripts that were classified into four main categories: 1) oxidation and reduction, 2) conjugation, 3) hydrolytic enzymes, and 4) other transcripts with function possibly related to stress. In each category, the most abundant subcategory is presented in Table 2. Among the oxidation and reduction enzymes, cytochrome p450 genes were the most abundant ones. Glycosyl transferases were the most abundant transcripts among the conjugation enzymes. Acetylcholine and carboxyl esterases were the most frequently found hydrolytic enzyme transcripts. Heat shock proteins were also found in substantial numbers.

**Table 2:** Detoxification genes expressed in the reference transcriptome.

Detoxification gene transcripts	Numbers found
**Oxidation and reduction enzymes**	**132**
Cytochrome P450s	85
**Conjugation enzymes**	**103**
Glycosyl transferases	37
**Hydrolytic enzymes**	**69**
Acetylcholine and carboxyl esterases	28
**Possible stress related functions**	**63**
Heatshock proteins	29

## Discussion

After cleaning and quality checks, we obtained 1.7 Gb of reads. *Nauphoeta cinerea* has a C value (pg) of 5.15, or a genome size of approximately 5,000 Mb (Koshikawa *et al.*, 2008). Hence, our transcriptome, disregarding isoforms, represented around 30% of the *N. cinerea* genome. Our library was enriched for mRNA sequences, and the reads obtained were 75 bp long, sequenced as pair-end. Thus, it was necessary to reconstruct full-length transcripts by transcriptome assembly. A transcriptome assembly encounters many challenges, among them differential expression of transcripts and alternative splicing (Grabherr *et al.*, 2011). In spite of these challenges, the comparison with other cockroaches transcriptomes (Zhou *et al.*, 2014, Chen *et al.*, 2015; Kim *et al.*, 2016) showed that we had obtained a good assembly with a relatively small amount of data using Trinity v2.2.0 (Table 1). An outstanding characteristic of the cockroach transcriptomes that we assembled was the low level of GC content. A high GC content is correlated to high recombination rate, and in insects genomes, the GC content is usually low, but can be heterogeneous (Kent *et al.*, 2012; Kent and Zayed, 2013).

Blastn was used to find similarities between *N. cinerea* and the other cockroach transcriptomes, and large structural proteins gave among the best results (Table S1). The *B. germanica* assembly has overall better Blast results, which may be a consequence of its higher N50 provided by that the Roche 454 sequencing method, which produces longer reads that can improve the assembly in complex regions (Martin and Wang, 2011). Here, we used as a strategy a small number of individuals for RNA extraction, which on the one hand, simplifies the assembly as a result of less genetic variations, but on the other rules out the possibility of performing any population analysis or searches for SSR markers and SNPs.

The transcriptome annotation showed that the most frequent GOs (Figure 1 and 2) are similar to other insect transcriptomes (Zhou *et al.*, 2014, Chen *et al.*, 2015; Wadsworth and Dopman 2015; Kim *et al.*, 2016; Zhang *et al.*, 2016). The annotation also revealed a high similarity of the *N. cinerea* sequences with the termite *Zootermopsis nevadensis* (Blattodea). While this can be related to the amount of sequences available in databases, the phylogenetic relationship between termites and cockroaches is still controversial (Legendre *et al.*, 2015).

Up-regulated genes were more frequent in fat body, confirming the versatility of this organ in insects (Arresse and Soulages, 2010), even when compared to a tissue set that contains sensory organs and central nervous system ganglia (Figure 2). In addition, there were only 5,921 common Blast results among these tissues, in a total of 39,553 Blast results (Figure 3B). These numbers reflect the big functional difference among head and fat body tissue. It is important to note that although the fat body had a lesser number of assembled transcripts, it had more up-regulated genes in comparison with the head tissue.

The individuals used to generate the transcriptomes had not been submitted to any specific stress condition. Consequently, the elevated number of genes related to biotic and abiotic stress in the differential expression analysis confirm the role of the fat body as an active detoxification organ (Figure 2). Our interest in detoxification genes is due to the growing use of *N. cinerea* as a potential model for toxicological biochemistry studies. In a study aiming to identify candidate genes for insecticide resistance in insecticide susceptible and resistant strains of *Anopheles gambiae,* no single body part (including the fat body) emerged as the key site of overtranscription of putative insecticide resistance genes (Ingham *et al.*, 2014). In contrast, our result indicate a quite different pattern, with genes up-regulated in the fat body compared to head tissue in specimens maintained in the laboratory. It is important to highlight that the heads and fat bodies used for RNA extraction were from different individuals. A more comprehensive study design involving multiple dissected tissues and individuals exposed to different stress conditions would facilitate the comprehension of the role of the fat body in biotic and abiotic stress responses. It is well known that the fat body and hemocytes are the major components of the innate immune response in insects. Signals resulting from such stimuli can activate the synthesis and secretion of antimicrobial peptides by the fat body (Tsakas and Marmaras, 2010). However, the metabolic response of the fat body to ROS and the activation of inflammation-associated signaling pathways remains to be determined (Gloire *et al.*, 2006).

In the reference assembly, we found many genes related to detoxification, in similar number to those found in transcriptomes of other insects (Xu, *et al.*, 2013), indicating that our assembly strategy was efficient. Consequently, the sequences obtained here are a valuable source for future studies of such genes in *N. cinerea*. Detoxification related cytochrome p450 transcripts were found in the highest number (85). The termite *Zootermopsis nevadensis* genome has 76 p450 genes (Terrapon *et al.*, 2014). In contrast, a search for detoxification and insecticide target genes in *B. germanica*, resulted in 163 p450-related genes (Zhou *et al.*, 2014). A similar search previously done on the midgut transcriptome of *P. americana* resulted in 31 P450 transcripts (Zhang *et al.*, 2016). It is important to note that these results were obtained in transcriptome data that can both subestimate and overestimate this diversity compared to genomic analyses.

In conclusion, we obtained a total of 24,980,364 reads and 57,928 genes, constituting a public database for gene identification and expression analysis in *N. cinerea.* The data presented here are a starting point to understand the fat body metabolism of *N. cinerea* based on nucleic acid sequences. In addition, our results attest to the multifunctionality of the fat body in insects.

## Supplementary material

The following online material is available for this article:

Table S1 - Blastn results among cockroaches transcriptomes.
