# Supplementary material for: De novo transcriptome assembly of the lobster cockroach Nauphoeta cinerea (Blaberidae)
Source: Genet Mol Biol. 2018 Jul 16;41(3):713–21. doi: 10.1590/1678-4685-GMB-2017-0264 (PMC6136372; doi:10.1590/1678-4685-GMB-2017-0264)
Supplement: Supplementary file 1 [file 1415-4757-GMB-1678-4685-GMB-2017-0264-suppl.pdf]

Supplementary Material to "*De novo* transcriptome assembly of the lobster cockroach *Nauphoeta cinerea* (Blaberidae)"

Table S1 – Blastn results among cockroaches transcriptomes ordered by greater *Bitscore*s.

Nauphoeta cinerea X *Blattella germanica*			*Nauphoeta cinerea* X *Periplaneta americana*		
Gene	E-value	Bit Score	Gene	E-values	Bit Scores
Myosin heavy chain	0	90.22	*Twitchin*	0	82.13
Sectrin alpha chain	0	83.02	*PB2 mRNA for RNA polymerase II second largest subunit*	0	83.25
Clathrin, heavy chain	0	82.91	*Elongation factor 1-alpha mRNA*	0	88.2
Dynein heavy chain, cytoplasmic	0	82.53	*Spectrin alpha chain*	0	83.68
Pre mRNA processing splicing factor 8	0	85.83	*Exportin-7*	0	84.47
Sodium/potassium-transporting ATPase subunit alpha	0	87.45	*Plexin-A4*	0	82.01
Splicing factor 3B subunit 1	0	84.1	*Pre mRNA processing splicing factor 8*	0	85.76
ATP-dependent RNA helicase dbp2-like	0	83.67	*Puromycin-sensitive aminopeptidase*	0	81.26
Protein lingerer	0	84.31	*Phosphatidylinositol-binding clathrin assembly protein*	0	82.6
Chromodomain-helicase-DNA-binding protein Mi-2 homolog	0	81.22	*Dystonin*		
